# Low-Input High-Molecular-Weight DNA Extraction for Long-Read Sequencing From Plants of Diverse Families

**DOI:** 10.3389/fpls.2022.883897

**Published:** 2022-05-19

**Authors:** Alessia Russo, Baptiste Mayjonade, Daniel Frei, Giacomo Potente, Roman T. Kellenberger, Léa Frachon, Dario Copetti, Bruno Studer, Jürg E. Frey, Ueli Grossniklaus, Philipp M. Schlüter

**Affiliations:** ^1^Department of Plant and Microbial Biology and Zurich-Basel Plant Science Centre, University of Zurich, Zurich, Switzerland; ^2^Department of Plant Evolutionary Biology, Institute of Biology, University of Hohenheim, Stuttgart, Germany; ^3^Department of Systematic and Evolutionary Botany and Zurich-Basel Plant Science Centre, University of Zurich, Zurich, Switzerland; ^4^Laboratoire des Interactions Plantes Microbes Environnement (LIPME), INRAE, Toulouse, France; ^5^Department of Method Development and Analytics, Agroscope, Wädenswil, Switzerland; ^6^Department of Plant Sciences, University of Cambridge, Cambridge, United Kingdom; ^7^Institute of Agricultural Sciences and Zurich-Basel Plant Science Centre, ETH Zürich, Zurich, Switzerland

**Keywords:** DNA extraction, DNA sequencing, nanopore sequencing, Circulomics, plant genome, ONT long read sequencing, PacBio, genome assembly

## Abstract

Long-read DNA sequencing technologies require high molecular weight (HMW) DNA of adequate purity and integrity, which can be difficult to isolate from plant material. Plant leaves usually contain high levels of carbohydrates and secondary metabolites that can impact DNA purity, affecting downstream applications. Several protocols and kits are available for HMW DNA extraction, but they usually require a high amount of input material and often lead to substantial DNA fragmentation, making sequencing suboptimal in terms of read length and data yield. We here describe a protocol for plant HMW DNA extraction from low input material (0.1 g) which is easy to follow and quick (2.5 h). This method successfully enabled us to extract HMW from four species from different families (Orchidaceae, Poaceae, Brassicaceae, Asteraceae). In the case of recalcitrant species, we show that an additional purification step is sufficient to deliver a clean DNA sample. We demonstrate the suitability of our protocol for long-read sequencing on the Oxford Nanopore Technologies PromethION^®^ platform, with and without the use of a short fragment depletion kit.

## Introduction

Long-read sequencing technologies have reshaped the research landscape of plant biology over the last few years. With the recent increase in sequencing read length, decrease in sequencing cost, and newly developed bioinformatics tools suitable for these technologies, *de novo* assembly of large and complex plant genomes of non-model species is now feasible (Jiao and Schneeberger, 2017; Kersey, 2019). This offers unprecedented opportunities to investigate genome structure and function, and focus on molecular and evolutionary questions in organisms that were previously inaccessible (Belser et al., 2018). We are now gaining a deeper understanding of genomic diversity, evolution, and gene function by sequencing more genomes at a higher resolution (Zhang et al., 2017; Chawla et al., 2021). Lately, the possibility to release high quality reference genome assemblies has led to initiatives such as the European Reference Genome Atlas (Formenti et al., 2022) or the Earth BioGenome Project, which “aims to sequence, catalog and characterise the genomes of all of Earth’s eukaryotic biodiversity,” to study evolution and preserve biodiversity (Exposito-Alonso et al., 2020).

Long-read sequencers are able to generate reads of 10 kbp or longer. The recently developed PacBio^®^ HiFi technology from Pacific Biosciences can provide reads up to 25 kbp (the older CLR mode provides longer but less accurate reads)^1^, while Oxford Nanopore Technologies^®^ (ONT^®^) nanopore sequencing technology provides the longest reads, up to the current record of 4.2 Mbp^2^. Such long reads are able to unambiguously capture complex and repetitive regions in plant genomes, allowing the exploration of genomic regions that were previously inaccessible (Belser et al., 2018; Goerner-Potvin and Bourque, 2018). This, together with their ability to resolve highly heterozygous regions, has enabled the assembly of large plant genomes at the chromosome level (Hu et al., 2019; Hasing et al., 2020; Pu et al., 2020; Niu et al., 2022).

Long reads are also reshaping the way we approach population genetic studies. Structural Variants (SVs) represent a major form of genetic variation, and may contribute to phenotypic variation as much as – or even more so than – single nucleotide polymorphisms (SNPs) (Chawla et al., 2021). However, it is challenging to reliably detect large SVs using short-read sequencing (Saxena et al., 2014). Structural variants can now be captured with long reads, thus enabling the fine-scale characterisation of genomic rearrangements responsible for trait variation in plants (Zhang et al., 2016; Sedlazeck et al., 2018; Heller and Vingron, 2019). Moreover, since PacBio^®^ and ONT^®^ are able to detect chemical modifications on nucleotides, they provide a new method to directly profile patterns of DNA methylation across genomes and allow epigenetic studies (Flusberg et al., 2010; Simpson et al., 2017). However, to fully exploit the potential of long-read sequencing, it is critical to obtain high molecular weight (HMW) DNA of adequate purity and integrity.

Extraction of HMW DNA from plant material can be challenging. First, plant cells have a cell wall composed of polysaccharide polymers, such as cellulose and pectin, as well as glycoproteins and lignin (Zhang et al., 2021), making the cell wall rigid and hard to break. Thus, steps that achieve effective mechanical disruption of the cell wall are necessary. As a chemical defence against herbivores, plants also produce polysaccharides and phenols, which tend to accumulate in leaves and, upon cell lysis, can bind DNA and affect downstream molecular analyses (Katterman and Shattuck, 1983; Varma et al., 2007; Moreira et al., 2011). The presence of polysaccharides has been shown to inhibit restriction enzyme activity (Pandey et al., 1996). Thus, purification of DNA from plant material requires careful optimisation. Several commercial DNA isolation kits and protocols are available on the market. Many protocols rely on the isolation of nuclei with an osmotic nuclear isolation buffer, and subsequent lysis of nuclear membranes with a detergent to release DNA. These methods are time-consuming, hazardous, and/or require high amounts of input material (Zerpa-Catanho et al., 2021). Furthermore, the resulting genomic DNA (gDNA) is often highly oxidised and, therefore, unsuitable for long-read applications. Kit-based extraction methods are offered by several companies, and are intended to easily remove contaminants; but they are costly and there is a risk of losing DNA during column washes. A previously published protocol (Mayjonade et al., 2016) presented a method to extract plant HMW DNA via a sodium dodecyl sulphate (SDS)-based lysis buffer and magnetic bead-based purification. The described method is easy and quick, taking only 1.5 h to complete DNA isolation from harvested plant material. Moreover, it requires only 0.1 g starting material. Unfortunately, this method did not yield DNA of sufficient quality for long-read sequencing in the plant species used as study organisms by our groups.

**FIGURE 1:**
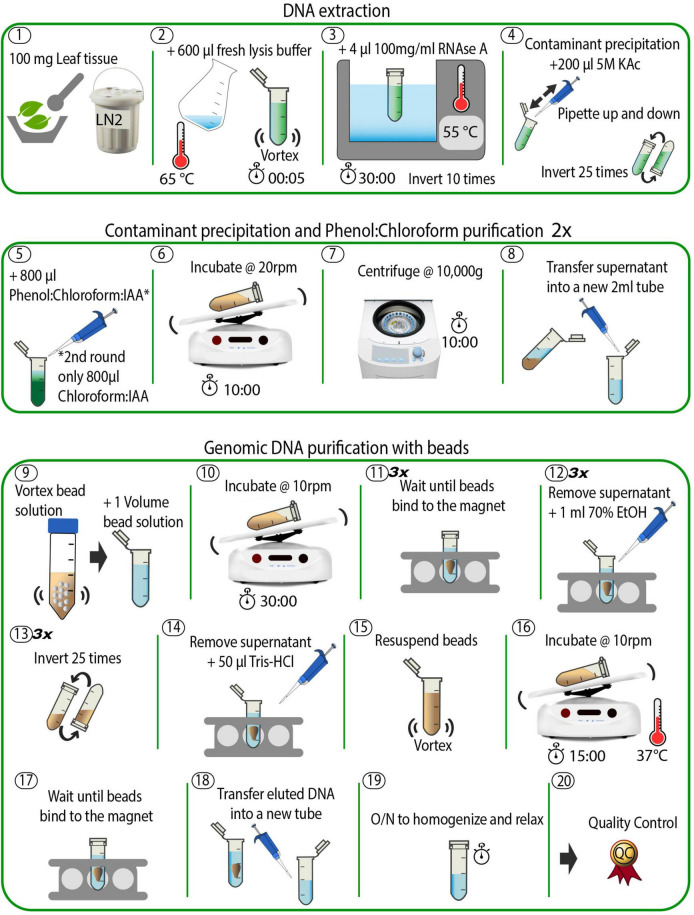
Schematic overview of the DNA extraction method. The individual steps are described in detail in the see section “Methods” and in the accompanying online protocol.

Here, we present an improved HMW DNA extraction method based upon the protocol by Mayjonade et al. (2016). We introduced two simple but effective major modifications: the addition of β-mercaptoethanol, which prevents oxidative damage to nucleic acids (Gerstein, 2001) and prevents nuclease activity, and a phenol:chloroform extraction. To demonstrate the robustness of the method, we applied it to plants from diverse families across both monocots and eudicots, including the Mediterranean early spider orchid (*Ophrys sphegodes*, Orchidaceae), Italian ryegrass (*Lolium multiflorum*, Poaceae), wild cabbage (*Brassica incana*, Brassicaceae), and South African beetle daisy (*Gorteria diffusa*, Asteraceae). We successfully extracted HMW DNA of high purity and integrity from all four species, showing that our protocol can be applied to a broad range of angiosperm species. To demonstrate the suitability of our protocol for long-read technology, we sequenced one sample each from *O. sphegodes* and *L. multiflorum* on the ONT^®^ PromethION^®^ platform. Finally, we assessed the use of Circulomics’ Short Read Eliminator Kit and its impact on sequenced read length in these species. We show that short fragment removal can improve the average read length and increase the proportion of ultra-long sequenced fragments [>100 kb (Prall et al., 2021)], thus improving sequencing efficiency. A schematic overview of our protocol is provided in Figure 1 and a step-by-step version is available at dx.doi.org/10.17504/protocols.io.5t7g6rn online.

## Methods

### Species Used for DNA Extraction

We used plants from four families for DNA extraction, specifically *Ophrys sphegodes* Mill. (Orchidaceae), *Lolium multiflorum* Lam. (Poaceae), *Brassica incana* Ten. (Brassicaceae) and *Gorteria diffusa* Thunb. (Asteraceae). Since bee orchids (*Ophrys*) hardly produce 2 g total leaf mass per individual, common DNA extraction procedures that need ≥ 1 g starting material make it difficult to reach a final DNA amount suitable for long-read sequencing without pooling individuals. This, coupled with the large genome size (ca. 1C = 5 Gbp) and high heterozygosity, would make a genome assembly project unfeasible. Italian ryegrass is one of the most important forage grasses (Gilliland et al., 2007) and represents a major feed source for livestock farming due to its high digestibility and biomass yield (Wilkins, 1991; Frei et al., 2021). The Brassicaceae family includes diverse plant species widely cultivated for oilseed production and vegetable consumption, including oilseed rape, kale, broccoli and cauliflower. The major challenge of extracting DNA from these plants comes from the high level of secondary metabolites in leaves that interfere with sample purity (Zhao et al., 2020). The leaves of *G. diffusa* are even more challenging and contain milky latex (and other unknown secondary metabolites) in high amounts.

### High Molecular Weight DNA Extraction for *Ophrys sphegodes*

Plant material used for this experiment was collected from a greenhouse-grown *O. sphegodes* individual. Young (2 weeks old) leaves were collected, flash frozen in liquid nitrogen (LN_2_), and stored at −80°C until DNA extraction. On the day of the experiment, a fresh SDS lysis buffer was prepared as in Mayjonade et al. (2016), supplemented with β-mercaptoethanol (β-ME): 1% polyvinylpyrrolidone 40 (PVP40), 1% sodium metabisulphite, 0.5 M sodium chloride, 100 mM Tris–HCl (pH 8), 50 mM EDTA (pH 8), 2% β-ME, 1.5% sodium dodecyl sulphate (SDS), in ddH_2_O to a final volume of 10 ml of stock solution (see Supplementary Table 1). The lysis buffer was incubated at 65°C for 1 h to ensure total dissolution of reagents in the buffer. Meanwhile, 100 mg frozen leaf tissue was ground with mortar and pestle (pre-cooled at −80°C for > 1 h) in LN_2_ until a fine powder was obtained (Step 1, Figure 1). Note that it is not advisable to use more than 100 mg starting material, as this decreases DNA purity (as measured by the A_260/230_ absorbance ratio using a NanoDrop^®^ spectrophotometer). Also, since the grinding step is crucial to ensure optimal outcomes in terms of final DNA yield and integrity, we provide some tips. First, it is critical to keep the sample submerged in LN_2_. If the LN_2_ evaporates, plant material will thaw (as indicated by a colour change – it becomes dark green when it thaws) and the DNA will degrade. Second, a fine, flour-like texture of ground plant material is optimal to ensure maximal DNA yield. Hence, the plant material was first crushed in a mortar by gently pounding it with a pestle, until small pieces (<5 × 5 mm) were obtained. Then, plant pieces were rubbed against the mortar with circular movements of the pestle to obtain a final powder with a flour-like texture. To avoid thawing, LN_2_ was added every half minute (or when LN_2_ had almost evaporated). Depending on the plant material and the pressure applied, the grinding can take up to 30 min. After grinding, the powder was immediately transferred to a sterile 2 ml plastic tube with a chilled metal spatula and mixed with 600 μl of the pre-warmed (65°C) SDS lysis buffer (Step 2 in Figure 1). The sample was vortexed for 3–5 s and incubated on a thermomixer with gentle agitation (400 rpm, 20 min at 55°C) to inactivate DNases and remove polyphenols that could bind DNA. Afterward, 4 μl of 100 mg/ml DNase-free RNase A (Qiagen, Germantown, MD, United States) were added, and the sample was incubated for 10 additional minutes at 55°C (Step 3, Figure 1). To fully precipitate proteins and polysaccharides that form complexes with SDS (Otzen, 2011), 200 μl of 5 M potassium acetate (KAc) were added, and the solution was mixed by inverting the tube 25 times (Step 4, Figure 1). Next, the sample was purified via a phenol/chloroform extraction as follows. Under a fume hood, 800 μl of a phenol:chloroform:isoamyl alcohol mixture (25:24:1 v/v, pH 8) was added, and the sample was incubated for 10 min at room temperature (RT) with gentle agitation on a tube rotator at 20 rpm (Steps 5 and 6, Figure 1). The sample was then centrifuged for 10 min at 10,000 × *g* at RT (Step 7, Figure 1). Afterward, the supernatant was transferred into a new 2 ml tube using a 1,000 μl wide-bore pipette tip, to avoid shearing the DNA (Step 8, Figure 1). A second purification step was then undertaken by the addition of 800 μl chloroform:isoamyl alcohol (24:1 v/v), followed by a second incubation (10 min at RT at 20 rpm) and centrifugation (10 min at 10,000 × *g* at RT). Finally, the supernatant was transferred to a new 2 ml tube and the final volume was recorded (∼ 700–800 μl).

### Carboxyl Magnetic Bead Purification

The supernatant recovered from the previous step was purified with carboxylated magnetic beads (Sera-Mag SpeedBeads*™* Carboxyl Magnetic Beads, GE Healthcare 65152105050250, Fisher Scientific). The bead stock solution was prepared as in Schalamun et al. (2019) and added to the sample in a 1:1 ratio (Step 9, Figure 1) to remove shorter fragments. Note that a complete resuspension of beads in the stock solution was crucial for optimal DNA yield. The sample tube was incubated for 30 min on a rotator at 10 rpm at RT, spun down for 1 s in a benchtop microcentrifuge, and placed into a magnetic rack until all beads migrated toward the magnet and the solution became clear (Step 10 and 11, Figure 1). This step can take several minutes, as the viscosity of the solution may slow down the beads’ migration. Afterward, an ethanol (EtOH) washing step was carried out as follows: 1 ml of 70% EtOH was added to the tube; then, the sample tube was removed from the magnetic rack (without extended incubation), mixed by inverting it 25 times to resuspend the bead pellet, spun down for 1 s, and placed back into the magnetic rack. When the solution became clear, the supernatant was discarded, and the washing step repeated for a total of three times (Step 12–14, Figure 1). We note that these steps can be quite challenging to perform, as beads tend to aggregate, making it difficult to separate and resuspend them properly. In this case, it helps to gently flick the tube to help the beads separate and to avoid prolonged incubation in EtOH. Thereafter, DNA was eluted by addition of 50 μl of 10 mM Tris–HCl pH 8.5 (using commercial buffer EB from Qiagen) preheated to 50°C to the tube, followed by a last incubation at 37°C for 15 min (Steps 14–16, Figure 1). The warm temperature is intended to favour the elution of DNA from the magnetic beads. Finally, to collect the eluted DNA, the tube was placed back into the magnetic rack until the solution became clear (Step 17, Figure 1). This step lasted ca. 30 min, as the long fragments migrate slowly in such a small volume. Note that the slow separation speed is an indication of successful HMW DNA extraction. The eluted DNA was gently pipetted into a new 2 ml tube with a wide-bore 1000 μl pipette tip (Step 18, Figure 1). The sample was very viscous at this point, indicating highly concentrated HMW DNA. The tube was left on the bench overnight at RT to allow DNA to homogenise and relax (Step 19, Figure 1). The next day, the sample was ready for quality control (Step 20, Figure 1). A total of six *O. sphegodes* samples were prepared (named OPH_1-6). The total amount of time taken for DNA extraction and clean-up was approximately 2.5 h.

### High Molecular Weight DNA Extraction for Other Plant Species

To evaluate the efficacy and reproducibility of the protocol, we applied it to plant material from four different families: Poaceae (Italian ryegrass, *Lolium multiflorum*), Brassicaceae (wild cabbage, *Brassica incana*) and Asteraceae (beetle daisy, *Gorteria diffusa*). When extracting HMW DNA from Italian ryegrass leaf material, we noticed that the ground powder easily clumped when in contact with SDS lysis buffer (Step 2, Figure 1), thereby impacting the efficiency of the lysis step. To avoid powder clumping, it was critical to vortex the sample tube immediately after transferring the powder into the pre-warmed SDS lysis buffer. A total of three DNA samples were extracted (named RAB_1-3).

Wild cabbage leaves contain high amounts of polysaccharides that negatively impact final DNA purity and yield. To reduce the level of polysaccharides in leaf tissues, the plant was placed in the dark for ca. 18 h before harvesting (final sample named BRI_1).

Milky latex and other unknown secondary metabolites are present in high amounts in *G. diffusa* leaves. Carryover of these substances negatively impacts sample purity, resulting in NanoDrop A_260/230_ ratios outside the optimal range for long-read sequencing. We noticed that *G. diffusa* plants perish quickly without light, making a prolonged dark treatment infeasible. To address these issues, we reduced the amount of starting material and included an additional purification step with magnetic beads. Starting with 70–80 mg young leaves, the protocol was performed as described before until the final DNA elution in 50 μl of 10 mM Tris–HCl buffer (pH 8.5) (Steps 1–18, Figure 1). A total of eight tubes were prepared. DNA from two tubes each were pooled and carried through an additional bead purification step. Briefly, 1 volume of magnetic bead solution was added to each sample, followed by incubation at RT for 10–15 min (Steps 9 and 10, Figure 1). The tubes were placed into the magnetic rack until the solution became clear, and three washing steps were performed with 1 ml of 70% EtOH without removing the tubes from the rack (Step 11–12, Figure 1). After the last wash, the tubes were spun down for 1 s and placed back on the magnetic rack. The beads were resuspended in 50 μl 10 mM Tris–HCl buffer (pH 8.5) (Step 14–15, Figure 1) and incubated at 37°C for 15 min. Afterward, the tubes were placed on a magnetic rack for final DNA elution (Step 16–19, Figure 1) (samples named GOR_1-4).

### Quality Control Prior to Sequencing

Genomic DNA was evaluated for purity on a NanoDrop^®^ spectrophotometer ND-1000 (Thermo Fisher Scientific, MA, United States). Absorbance at 230, 260, and 280 nm was measured, and A_260/280_ and A_260/230_ ratios were assessed to determine DNA purity. Genomic DNA concentration was measured via NanoDrop^®^ and confirmed with a Qubit^®^ 3.0 fluorometer (Invitrogen, CA, United States) using the dsDNA BR Assay Kit (Thermo Fisher Scientific, MA, United States, Q32850). Note that, if the DNA is pure, the measurements of DNA concentrations from NanoDrop^®^ and Qubit^®^ should be identical.

DNA integrity was assessed on a TapeStation 4200 system (Agilent, CA, United States) with a Genomic DNA ScreenTape Assay. Here, DNA quality is assessed using a DNA Integrity Number (DIN) that ranges from 1 (highly degraded DNA) to 10 (intact DNA). Fragment length was measured on a Femto Pulse v-1.0.0.32 system (Agilent, CA, United States, Cat N° M5330AA) using the Genomic DNA 165 kb Ladder Fast Separation assay with a separation time of 70 min (Agilent, CA, United States, Cat N° FP-1002-0275). For *G. diffusa*, DNA integrity was determined on a Pippin Pulse*™* electrophoresis system (Sage Science, MA, United States, Cat N° PP10200) with program 5–80 kb and the Bio-Rad CHEF 5 kb DNA Size Standard.

### Size Selection With the Circulomics Kit

We tested the impact of short DNA fragment depletion on final sequencing results by applying the Circulomics Short Read Eliminator Kit (Circulomics, MD, United States, SS-100-101-01) on one of the two samples selected for sequencing (OPH_3 and RAB_2). The kit was applied to the *L. multiflorum* sample RAB_2 before library preparation to remove small DNA fragments. According to supplier information, the kit uses size-selective precipitation to reduce the amount of DNA fragments below 25 kbp in length^3^. Potentially, the kit can thus significantly enhance average read length during sequencing. The kit was used according to the manufacturer’s recommendations (handbook v2.0, 07/2019). Briefly, 60 μl of Buffer SRE were added to the sample tube (60 μl volume), gently mixed and the tube centrifuged at 10,000 × *g* for 30 min at RT. After supernatant removal, two washing steps were performed with 200 μl of 70% EtOH and a centrifugation at 10000 × *g* for 2 min at RT. Finally, 100 μl Qiagen Buffer EB were added and the tube was incubated at RT overnight to ensure efficient DNA elution (sample named RAB_2_Circ). No Circulomics kit was applied to sample OPH_3.

### ONT^®^ Library Preparation

We tested the suitability of our protocol by sequencing HMW DNA from samples OPH_3 and RAB_2_Circ. Sequencing library preparation was carried out following the general guidelines from Oxford Nanopore Technologies^®^ for 1D Genomic DNA sequencing, with modifications proposed by New England Biolabs^®^ (NEB) to ensure high data yield production and long-fragment sequencing. For library preparation, the following reagents were used: Ligation Sequencing Kit SQK-LSK109 (Oxford Nanopore Technologies^®^), NEBNext^®^ Companion Module for Oxford Nanopore Technologies^®^ Ligation Sequencing (NEB, MA, United States, Cat N° E7180S), and AMPure XP beads (Beckman Coulter Inc., CA, United States). A DNA amount of 1.5 μg was collected from samples OPH_3 and RAB_2_Circ (since OPH_3 was highly concentrated, 13 μl of DNA were diluted in 35 μl of 10 mM Tris–HCl buffer to reach a total volume of 48 μl) and transferred into a 0.2 ml thin-walled PCR tube. DNA fragments were repaired and end-prepped as follows: 3.5 μl NEBNext^®^ FFPE DNA Repair Buffer, 2 μl NEBNext^®^ FFPE DNA Repair Mix, 3.5 μl NEBNext^®^ Ultra*™* II End Prep Reaction Buffer, and 3 μl NEBNext^®^ Ultra*™* II End Prep Enzyme Mix were added to each tube. After mixing and spinning down, the samples were incubated at 20°C for 30 min, followed by a second incubation at 65°C for 30 min. The original recommendations from NEB were followed, instead of the ONT^®^ guidelines, as preliminary experiments showed better results under NEB supplier conditions: prolonged incubation time allowed recovery of longer fragments. After incubation, the solution from each tube was transferred to a clean 1.5 ml Eppendorf DNA LoBind^®^ tube (Eppendorf AG, Hamburg, Germany) for clean-up. First, a stock solution of AMPure XP Beads was prepared as in Schalamun et al. (2019), and 60 μl were added to each tube. The samples were then incubated on a HulaMixer™ sample mixer (Thermo Fisher Scientific, MA, United States, 15920D) for 20 min at RT, until the solution was homogenised. Bead clean-up was performed with two washing steps on a magnetic rack, each time pipetting off the supernatant and adding 200 μl of freshly prepared 70% EtOH. The pellet was resuspended in 61 μl nuclease-free water (1 μl was then taken out for quantification) and incubated for 10 min at RT on a HulaMixer™. Tubes were placed on a magnetic rack to collect the final eluate. For adapter ligation and clean-up, 60 μl DNA from the previous step was combined with 25 μl Ligation Buffer LNB, 5 μl Adapter Mix AMX, and 10 μl NEBNext^®^ Quick T4 DNA Ligase (240 μl) in a 1.5 ml Eppendorf DNA LoBind tube, and incubated for 20 min at RT. A second AMPure bead clean-up step was carried out by adding 45 μl of bead solution to each tube, followed by incubation on a HulaMixer™ for 20 min at RT. After pipetting off the supernatant on a magnet rack, the beads were washed twice with 250 μl Long Fragment Buffer LFB. Finally, the supernatant was discarded, the pellet resuspended in 25 μl Elution Buffer EB, and incubated for 10 min at RT to collect the final library.

### Long-Read Sequencing

The ONT^®^ PromethION PTC0031 sequencing platform (Alpha-Beta model, OS Ubuntu 16.06, Intel CPU) was used to sequence samples OPH_3 and RAB_2_Circ. For each sample, 0.8–1 μg of the prepared library was loaded onto a R9.4.1 chemistry PromethION flow cell. Sequencing ran until the flow cell buffer was exhausted (63.10 h for OPH_3, 40.14 h for RAB_2_Circ). MinKNOW v-2.1 was used for data acquisition, real-time analysis and sample tracking. Basecalling was performed with Guppy v-3.0.4.

## Results

### DNA Purity and Quantity

**FIGURE 2:**
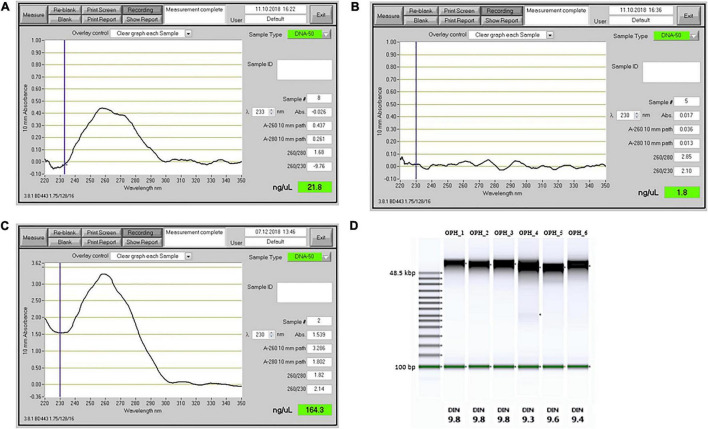
Comparison of DNA extraction performance for different extraction methods. **(A–C)** Output from a NanoDrop spectrophotometer. **(A)** DNA extraction results with the Mayjonade et al. (2016) protocol, without β-ME and phenol:chloroform purification step. **(B)** DNA extraction with lysis buffer as described by Schalamun et al. (2019). **(C)** DNA extracted with the protocol described in this study (sample OPH_3). **(D)** TapeStation results showing the fragment size distribution and the DNA Integrity Number (DIN), for six *O. sphegodes* samples.

We compared our DNA extraction protocol with the original version of Mayjonade et al. (2016) and the protocol of Schalamun et al. (2019) which was developed for recalcitrant plant species. We used *O. sphegodes* leaves and compared the results in terms of purity on a NanoDrop^®^ device. When using the original protocol (Mayjonade et al., 2016), we could recover only small amounts of DNA (1,090 ng DNA total) and failed to recover pure DNA (Figure 2A). The A_260/280_ = 1.68 indicated protein contamination, and the low A_260/230_ value indicated presence of polysaccharides, polyphenols or other organic compounds. Using the protocol of Schalamun et al. (2019), we were not able to recover any DNA at all (Figure 2B). With our improved extraction protocol, all purity criteria (A_260/280_ = 1.80–2.0 and A_260/230_ = 2.0–2.2) were met in all six *O. sphegodes* samples (Figure 2C, OPH_1-6 in Table 1). Remarkably, we were able to recover an average DNA amount of 5,710 ng per sample (3,950 to 8,450 ng). The NanoDrop^®^ and Qubit^®^ measurements were comparable (NanoDrop^®^/Qubit^®^ ratio close to 1; Table 1), indicating a clean DNA sample. Since Qubit^®^ measures only double-stranded DNA, a ratio of 1 further indicates that the DNA molecules measured are double stranded and that other molecules with absorption at 260 nm are absent (O’Neill et al., 2011). When the ratio was below 1 (OPH_2 = 0.87, OPH_5 = 0.71), we considered the Qubit^®^ values to be more accurate.

**TABLE 1:** Summary of DNA quality measurements for all extracted samples.

Species/Sample	Qubit conc. [ng/μl]	NanoDrop conc. [ng/μl]	Output [μg]	A_260/280_ ratio	A_260/230_ ratio	NanoDrop/Qubit conc. ratio	Qubit conc. after Circulomics [ng/μl]
*Ophrys sphegodes*							
OPH_1	79.0	114.4	3.95	1.83	1.97	1.45	–
OPH_2	115.0	99.8	5.75	1.83	2.23	0.87	–
OPH_3	169.0	164.3	8.45	1.82	2.14	0.97	–
OPH_4	86.3	85.9	4.32	1.81	2.15	0.99	–
OPH_5	152.0	107.9	7.60	1.79	1.97	0.71	–
OPH_6	83.8	86.4	4.19	1.79	1.79	1.03	–
*Lolium multiflorum*							
RAB_1	42.0	40.0	2.10	1.95	2.06	0.95	17.0
RAB_2	56.0	53.0	2.80	1.92	2.15	0.95	23.0
RAB_3	46.0	42.0	2.30	1.85	2.30	0.91	18.0
*Brassica incana*							
BRI_1	150.0	146.0	7.50	1.85	2.19	0.97	–
*Gorteria diffusa* ^1^							
GOR_1	146.0	182.6	7.30	1.84	2.07	1.25	–
GOR_2	93.6	97.0	4.68	1.83	2.18	1.04	–
GOR_3	102.0	108.4	5.10	1.82	2.15	1.06	–
GOR_4	103.0	112.8	5.15	1.82	2.12	1.09	–

*^1^For G. diffusa, 70–80 mg of input material were used, whereas for the other species, it was ∼100 mg.*

To evaluate the efficacy and reproducibility of our method, we applied it to other plant species. All three Italian ryegrass samples met the quality criteria, and the DNA amount recovered was on average 2,400 ng, with a NanoDrop^®^/Qubit^®^ ratio of ∼ 1 (RAB_1-3, Table 1). When DNA was extracted from *B. incana* leaves, A_260/280_ was 1.85 and A_260/230_ was 2.19. Total DNA amount was 7,300 ng, confirmed by a NanoDrop^®^/Qubit^®^ = 0.97 (Table 1). In *G. diffusa*, we included an additional purification step with magnetic beads after DNA elution. This extra step allowed all samples to meet the purity criteria necessary for sequencing (GOR_1-4, average DNA amount 5,550 ng per sample, A_260/280_ = ∼1.83, A_260/230_ = ∼2.13, Table 1).

### DNA Integrity and Fragment Lengths

**FIGURE 3:**
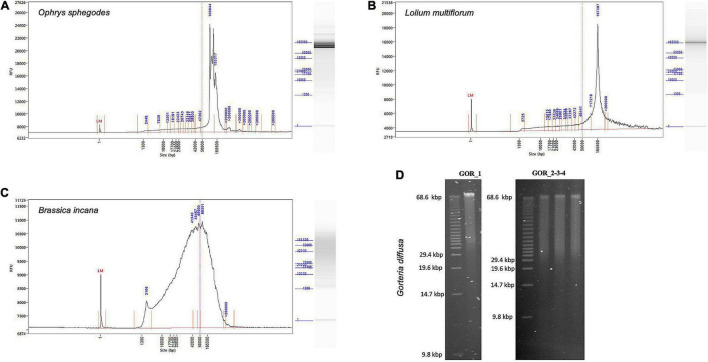
Fragment length profiles of extracted HMW DNA. **(A–C)** Femto Pulse profiles showing integrity and size of gDNA. **(A)** *O. sphegodes* sample OPH_3 with peaks at 110 and 153 kbp. **(B)** *L. multiflorum* (sample RAB_2) with a peak at 167 kbp. **(C)** *B. incana* (sample BRI_1) showing a broader fragment size distribution centred at 50 kbp. **(D)** Gel pictures for *G. diffusa* DNA extracts (GOR_1-4, from a pool of two initial extractions).

Genomic DNA integrity was assessed by determining the degree of fragmentation of the sample. On a TapeStation, all *O. sphegodes* samples had a DIN value of 9.3 or above (with 10 = highly intact DNA, Figure 2D). Fragment lengths of samples OPH_3, RAB_2 and BRI_1 were measured on a Femto Pulse system. Because the fast separation assay was used, it was not possible to distinguish any fragment lengths above 165 kbp, where only a compression band was visible. OPH_3 showed peaks at 110 and 153 kbp, and RAB_2 clearly displayed a peak at around 165 kbp (Figures 3A,B). BRI_1 showed a peak at around 50 kbp and a more heterogeneous distribution of fragment sizes (Figure 3C). DNA of GOR samples showed a clear band above the size of the largest marker of 68 kbp on a Pippin Pulse gel (Figure 3D).

### Size Selection With Circulomics and PromethION Sequencing

**FIGURE 4:**
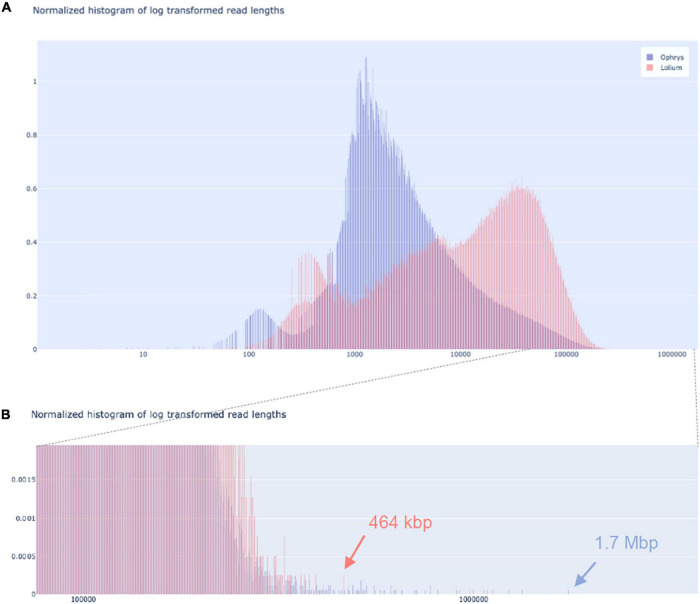
Nanopore sequencing read length distributions. Read length distribution for *O. sphegodes* OPH_3 (blue; without Circulomics kit) and *L. multiflorum* RAB_2_Circ (red; after Circulomics kit), showing **(A)** the entire normalised histogram of log-transformed read lengths and **(B)** a zoom-in into the section showing the longest reads (OPH_3 = 1.7 Mbp; RAB_2_Circ = 464 kbp).

We tested our DNA extraction protocol by sequencing samples OPH_3 and RAB_2_Circ. Each library was injected into one flow cell. Flow cell behaviour was comparable, with 2,791 and 2,684 out of 3,000 active channels for OPH_3 and RAB_2_Circ, respectively. Since the sequencing run was longer for OPH_3, the final sequencing yield in terms of Gbp data produced was higher for OPH_3 (66 Gbp versus 50 Gbp for RAB_2_Circ; Table 2). The effect of the Circulomics Kit in depleting short fragments is evident when comparing the fragment size distribution of the samples before (Figures 3A,B) and after (Figure 4) sequencing. When DNA molecules from samples OPH_3 and RAB_2 were evaluated on a Femto Pulse system, both showed the presence of smaller fragments (2–10 kbp). After short read depletion in sample RAB_2_Circ, the amount of small fragments decreased drastically, and as a result, the number of sequenced small reads was appreciably lower than in OPH_3 (Figure 4A and Table 2). Because short fragments were largely removed in RAB_2_Circ, more than half (51.84%) of the total reads were longer than 10 kbp, and one third (33.08%) were longer than 25 kbp. The read length statistics in OPH_3 were very different, with only 15.75% of the total reads longer than 10 kbp, and a read length N50 roughly half that of RAB_2_Circ (27,196 vs. 51,861 bp, Table 2). In both runs, we were able to recover long to ultra-long reads (>100 kbp) (Table 2). The longest reads were 464 kbp for RAB_2_Circ, and 1.7 Mbp for OPH_3.

**TABLE 2:** Nanopore (PromethION) sequencing statistics for sequenced samples.

Sample	OPH_3	RAB_2_Circ
Species	*O. sphegodes*	*L. multiflorum*
Treatment	No Circulomics	With Circulomics
Run duration (h)	63.10	40.14
Active channels	2,791	2,684
Total reads	9,131,684	2,181,501
Sequencing yield (Gbp)	66.4	50.4
Read length N50 (bp)	27,196	51,861
Mean read length (bp)	7,270.60	23,122.70
Median read length (bp)	2,157.00	11,119.00
# Reads ≥ 10 kbp	1,438,821	1,130,927
# Reads ≥ 25 kbp	635,963	721,668
# Reads ≥ 50 kbp	261,570	331,789
# Reads ≥ 100 kbp	55,592	59,998
# Reads ≥ 200 kbp	2,011	1,204
# Reads ≥ 500 kbp	39	0
% Reads ≥ 10 kbp	15.75%	51.84%
% Reads ≥ 25 kbp	6.96%	33.08%
% Reads ≥ 50 kbp	2.86%	15.21%
% Reads ≥ 100 kbp	0.61%	2.75%
% Reads ≥ 200 kbp	0.02%	0.06%
% Reads ≥ 500 kbp	0.00%	0.00%
Longest read (Mbp)	1.7	0.464

## Discussion

Long-read sequencing technologies offer a new array of opportunities to study plant genomes in ways that were not feasible before. Our study presents an improved HMW DNA extraction method suitable for a wide variety of plant species and shows how pure high-quality DNA, together with optimised library preparation parameters and size selection, are key for reaching high-throughput ultra-long reads for sequencing projects. Isolated DNA suitable for long-read sequencing has to meet two important criteria: (i) DNA purity and quantity and (ii) DNA integrity.

### DNA Purity and Quantity

Nucleic acid purification from plant tissue can be challenging. In particular, when chemicals such as ethanol or phenol are involved in a DNA extraction procedure, or when proteins or other organic components from plant material are present, final nucleic acid purity can be compromised. As a result, the purity of DNA extracts needs to be measured to avoid sequencing samples of inferior quality. The purity of isolated nucleic acids is commonly determined with a spectrophotometer, measuring three UV absorbance (A) values: absorbance at 260, 280, and 230 nm. Nucleic acids absorb UV light at a wavelength with a peak at 260 nm and hence an absorbance spectrum with a 260 nm peak indicates pure DNA. Protein (specifically the aromatic amino acids tryptophan and tyrosine) and phenols absorb UV light at 280 nm. When proteins and phenols contaminate a nucleic acid sample, the absorbance peak at 280 nm decreases by ca. 10-fold (Koetsier and Cantor, 2019). Organic components such as carbohydrates, buffer salts from DNA extraction (like Tris), EtOH, and EDTA strongly absorb at 230 nm. If one or more of these compounds are present in a nucleic acid extract, a lower 230 nm absorbance is detected. Overall, the ratios of the absorbance values A_260/280_ and A_260/230_ allow estimation of the purity of a nucleic acid sample. Pure dsDNA has absorbance ratios of A_260/280_ = 1.8 – 2.0 and A_260/230_ = 2.0 – 2.2; lower values indicate the presence of contaminants, while higher A_260/280_ values can be indicative of RNA contamination (pure RNA has an A_260/280_ = 2.0 – 2.2) (Glasel, 1995).

To obtain pure genomic DNA that meets the aforementioned characteristics, we first tried the protocols of both Mayjonade et al. (2016) and Schalamun et al. (2019) on *Ophrys* leaves, and then produced an improved and more robust method. The DNA extraction protocol from Mayjonade et al. (2016) is divided into three main steps: cell membrane disruption with SDS lysis buffer, contaminant precipitation with 5 M KAc, and final purification of gDNA with Sera-Mag SpeedBeads magnetic beads. Schalamun et al. (2019) introduced changes in lysis buffer composition, and used different incubation times at different temperatures. When our *Ophrys* sample was extracted with the protocol of Mayjonade et al. (2016), DNA concentration was low, and the UV absorbance spectrum showed severe contamination. A_260/280_ and A_260/230_ are unreliable at DNA concentrations < 20 ng/μl (Koetsier and Cantor, 2019), and in general such weakly concentrated samples are not suitable for long-read sequencing (as per manufacturers’ protocols; Figure 2A).

To produce a DNA extract of high concentration and purity, we modified the lysis buffer by increasing SDS concentration to 1.5% and adding 2% β-ME. A higher SDS concentration ensures a more effective rupture of cell walls after a first mechanical breakage by grinding under LN_2_, while β-ME is a reducing agent that denatures proteins by breaking the disulphide bonds between cysteine residues. Together, those two reagents increased the recovery of DNA from cells. Moreover, it has been reported that high levels of β-ME successfully remove polyphenols (Khanuja et al., 1999) and other organic compounds, such as tannins, from plant tissue (John, 1992; Moreira et al., 2011). A second modification was introduced after protein precipitation with 5M KAc. We added a phenol:chloroform:isoamyl alcohol (25:24:1 v/v) purification to remove other carryover contaminants. The separation of the solution in two phases allowed us to extract the isolated DNA in the aqueous phase, while leaving carryover contaminants from the extraction in the organic phase. Residual phenol was then removed during the bead purification step. These changes proved to be effective in delivering highly purified DNA in all our study species (see absorbance ratios in Table 1). All samples met the A_260/280_ and A_260/230_ criteria suitable for long-read sequencing, regardless of the plant species used. Remarkably, final DNA amounts were on average ∼ 5,000 ng per sample (from Qubit^®^ measurement; ∼100 ng/μl) and concentrations ranged from 42 to 169 ng/μl, depending on the species (Table 1). Hence, another positive effect of the improvements was the relatively high DNA quantity we were able to recover. ONT^®^ recommends using at least 1 μg DNA per library preparation. Thus, the final DNA amount per sample was enough for several parallel library preparations. Since PacBio^®^ recommends using 5 μg DNA for CLR and CCS sequencing (less if the plant genome of interest is 500 Mbp – 1 Gbp in size), sample pooling would have been necessary for sequencing with this platform.

The same DNA extraction procedure may result in different yields in different plant species, as different plants have different tissue characteristics. If the extracted DNA appears to be suboptimal in terms of purity, we suggest reducing the amount of starting material to 70–80 mg per sample, as we did with *G. diffusa*. In this way, the level of contaminants from plant material that may interfere with SDS during cell lysis is reduced. An additional purification step with magnetic beads can clean the DNA further. As long fragments bind to the magnetic beads and are not washed away, DNA yield is hardly impacted, while residual contaminants are removed. It may often be advisable to use softer and younger rather than older and tougher leaves. In this regard, plant secondary metabolites that negatively impact DNA extraction have been shown to accumulate over time as leaves age (Boege and Marquis, 2005; Moreira et al., 2011), although in other cases higher concentrations of phenolics have been found in young leaves (Barton et al., 2019). It is important to stress that the grinding step is crucial to recover a high DNA yield and that insufficient grinding can reduce final DNA yield. Finally, heating the elution buffer to 37°C before usage can help to increase the elution efficiency in the final step of the protocol.

### Integrity of DNA Molecules and Effect on Sequencing

In long-read sequencing, the fraction of long reads (10 kbp or longer) depends strongly on the integrity (i.e., degree of fragmentation) of the DNA molecules used for library preparation. Therefore, one of the goals of an HMW DNA extraction method suitable for long-read sequencing is to preserve and maximise long and ultra-long DNA molecules. One critical step is to prevent DNA damage by thawing. When harvesting, plant material should immediately be flash-frozen, stored at −80°C, and transferred to a cool mortar only before immediate use. During grinding, LN_2_ should not evaporate completely, as this can thaw plant material and cause DNA damage. Final extracted DNA can be stored at 4°C if actively used. For long-term storage, gDNA is best stored at −20°C and only thawed when used, as repeated cycles of freezing and thawing can degrade DNA molecules. Another source of DNA degradation is nuclease activity. During mechanical tissue rupture and chemical cell lysis, enzymes such as DNases are released along with DNA. For this reason, β-ME was added to the lysis buffer, which disrupts disulphide bonds, thus inactivating DNases (Price et al., 1969). For best results, we recommend to quickly homogenise the ground tissue powder immediately after adding lysis buffer by vortexing the sample tube. To prevent DNA fragmentation, it is also advisable to use wide bore pipette tips (or P1000 tips with cut ends). Such tips have a wider opening for aspirating and dispensing viscous solutions, and thus they can decrease DNA shearing due to pipetting. For the same reason, we advise carefully pipetting the DNA with slow, gentle movements of the pipette plunger. Since elution time plays a role in long fragment recovery, we prolonged the elution time during bead purification and library preparation. This helped recovering a higher fraction of long fragments and an overall higher DNA amount. As shown in Figure 4B and Table 2, these improvements allowed us to obtain long to ultra-long reads in both sequencing runs.

Finally, we recommend to remove short DNA molecules (<10 kbp) before sequencing. The presence of these “short” fragments does not result in poorer quality sequence, but it does affect a sequencing run’s fragment size distribution (cf. Figure 4). Short fragments compete with longer ones for pore occupancy in the flow cell, decreasing the throughput of long reads per sequencing run. Although shorter reads can be discarded bioinformatically, this approach may not be optimal in terms of cost efficiency when the aim of long-read sequencing is to generate data for genome assembly.

## Conclusion

Long-read sequencing approaches are starting to dominate as the primary tools for genome sequencing projects. Thanks to continuous improvements in sequencing chemistry and technology, long reads are used in a plethora of applications, from *de novo* genome assembly to methylome analysis, to field applications. Here, we provide a robust method to extract purified high molecular weight DNA for long-read sequencing. Our DNA extraction method successfully worked on diverse monocot and eudicot plant species, indicating that the method is effective in a wide variety of plant families. For particularly challenging species, we show that adding an extra purification step allows the user to achieve the purity required for long-read sequencing, while maintaining a high integrity of the DNA molecules. A step-by-step version of the protocol is available online at dx.doi.org/10.17504/protocols.io.5t7g6rn.

## Data Availability Statement

The original contributions presented in the study are included in the article/Supplementary Material, further inquiries can be directed to the corresponding authors.

## Author Contributions

AR and PS: conceptualisation and writing—original draft. AR, BM, GP, and DF: methodology and investigation. RK, LF, and DF: validation. AR: data curation and visualisation. PS and UG: supervision. DC, BS, JF, UG, and PS: resources. BS, JF, UG, and PS: funding acquisition. All authors: writing, review, and editing.

## Conflict of Interest

The authors declare that the research was conducted in the absence of any commercial or financial relationships that could be construed as a potential conflict of interest.

## Publisher’s Note

All claims expressed in this article are solely those of the authors and do not necessarily represent those of their affiliated organizations, or those of the publisher, the editors and the reviewers. Any product that may be evaluated in this article, or claim that may be made by its manufacturer, is not guaranteed or endorsed by the publisher.

## Footnotes

1 https://www.pacb.com/literature/application-brief-whole-genome-sequencing-for-de novo-assembly-best-practices/

2
https://nanoporetech.com/about-us/news/ncm-announcements-include-single-read-accuracy-991-new-chemistry-and-sequencing

3
https://www.circulomics.com/store/Short-Read-Eliminator-Kit-p131401036
