# Supplementary material for: Low-Input High-Molecular-Weight DNA Extraction for Long-Read Sequencing From Plants of Diverse Families
Source: Front Plant Sci. 2022 May 19;13:883897. doi: 10.3389/fpls.2022.883897 (PMC9161206; doi:10.3389/fpls.2022.883897)
Supplement: Supplementary file 1 [file Table_1.DOCX]

# SUPPLEMENTARY INFORMATION

## Table 1S. Lysis buffer

| **Reagent** | **Stock concentration** | **Final amount** | **Final concentration** |
| --- | --- | --- | --- |
| PVP40 |  | 0.1 g | 1% |
| Sodium metabisulphite |  | 0.1 g | 1% |
| NaCl | 2.5 M | 2 ml | 0.5 M |
| Tris HCl pH 8 | 1 M | 1 ml | 100 mM |
| EDTA pH 8 | 0.5 M | 1 ml | 50 mM |
| β-mercaptoethanol |  | 200 µl | 2% |
| SDS | 20% | 750 µl | 1.5% |
| H_2_O (mol. biol. grade) |  | Up to 10 ml |  |
